# Sex-Biased Expression of Genes Allocated in the Autosomal Chromosomes: Blood LC-MS/MS Protein Profiling in Healthy Subjects

**DOI:** 10.1155/2023/8822205

**Published:** 2023-03-11

**Authors:** Hayder A. Giha, Rabab A. Abdulwahab, Jaafar Abbas, Zakia Shinwari, Ayodele Alaiya

**Affiliations:** ^1^Department of Medical Biochemistry, College of Medicine and Medical Sciences, Arabian Gulf University, Manama 26671, Bahrain; ^2^Medical Biochemistry and Molecular Biology, Khartoum, Sudan; ^3^Integrated Sciences Department, College of Health and Sport Sciences, University of Bahrain, Manama 32038, Bahrain; ^4^Al Jawhara Centre for Molecular Medicine and Inherited Disorders, Arabian Gulf University, Manama 26671, Bahrain; ^5^Arad Health Center, Muharraq, Bahrain and Gulf Medical and Diabetes Center, Manama, Bahrain; ^6^Proteomics Unit, Stem Cell and Tissue Re-Engineering Program, King Faisal Specialist Hospital and Research Centre, Riyadh 11211, Saudi Arabia

## Abstract

**Background:**

Sex and gender have a large impact in human health and disease prediction. According to genomic/genetics, men differ from women by a limited number of genes in Y chromosome, while the phenotypes of the 2 sexes differ markedly.

**Methods:**

In this study, serum samples from six healthy Bahraini men and women were analyzed by liquid chromatography–mass spectrometry (LC-MS/MS). Bioinformatics databases and tools were used for protein/peptide (PPs) identification and gene localization. The PPs that differed significantly (*p* < 0.05, ANOVA) in abundance with a fold change (FC) of ≥1.5 were identified.

**Results:**

Revealed 20 PPs, 11 were upregulated in women with very high FC (up to 8 folds), and 9 were upregulated in men but with much lower FC. The PPs are encoded by genes located in autosomal chromosomes, indicative of sex-biased gene expression. The only PP related to sex, the sex hormone-binding globulin, was upregulated in women. The remaining PPs were involved in immunity, lipid metabolism, gene expression, connective tissue, and others, with some overlap in function.

**Conclusions:**

The upregulated PPs in men or women are mostly reflecting the functon or risk/protection provided by the PPs to the specific sex, e.g., Apo-B100 of LDLC. Finally, the basis of sex-biased gene expression and sex phenotypic differences needs further investigation.

## 1. Introduction

Biological and physiological differences exist between men and women [1]. The conventional molecular distinctions between the two sexes are mainly the genomic makeup and transcriptomic typing [2, 3], while the proteomic differences are persuaded by epigenetics, alternative splicing, posttranslation modifications, as well as others [4]. However, the influence of sex hormones on gene expression should not be overlooked. A new and expanded central dogma composed of OMICs platform, constituted of genome, transcriptome, and proteome, is needed for better utilization of the human genome data. Although the human genome is expected to constitute between 20,000 and 25,000 genes [5], more than 2 million proteins and peptides (PPs) are detected by proteomics [6]. In addition to their normal physiological roles, PPs are central players in disease etiology, pathology, and complications as well as they are utilized as diagnostic and prognostic biomarkers [7]. 

In the era of personalized and precision medicine, sex (biological determinants of men and women) and gender (environmental and cultural determinants) stand early during the diversification of people into categories and subcategories down to the level of individuals. Therefore, an initial step towards the personalization of medicine would be setting the molecular differences between men and women. The inherent susceptibility of men and women to disease is known to vary considerably, e.g., men are more prone to infections, coronary heart disease (CHD), while women are more prone to autoimmune and inflammatory diseases [8–11]. Moreover, it is well known that women have more vigorous innate immunity [9] and humoral immunity to antigenic challenges [12], which accelerate pathogen clearance but can lead to an increased frequency of immunological disorders such as autoimmune or inflammatory diseases [10, 13]. Moreover, it has been observed that sex/gender affects the responses to vaccination and its outcome [14]. Finally, women's pregnancy is another determinant for immune and inflammatory responses [15].

Proteomics is an up-and-coming discipline emerged from the Human Genome Project and became an indispensable platform for a better understanding of genomic and transcriptomic data [16]. Liquid chromatography–mass spectrometry (LC-MS/MS) is the major tool that has revolutionized the proteomic analysis [17]. Complimented with bioinformatics, the proteomics resolved the issue of protein/peptide detection, quantitation, and together with sequence identification, they facilitate protein structure and function prediction and their possible roles in health and disease. Moreover, the proteomic analysis is superior to the other biochemical and antibody-based approaches in the ability to detect with high accuracy and sensitivity the small differences in PPs in study materials [18]. Lastly, the used techniques and software are regularly upgraded for the generation and analysis of larger data [19].

Usually, studies of the differences between men and women are focused on physiological events, susceptibility to a specific disorder, drug response/interaction, or variations of biochemical markers [20]. The sex-specific physiological events or disorders, e.g., pregnancy and lactation or breast and prostatic tumors, were organ-specific with different markers and susceptibility factors [21]. However, only very few studies have focused on the differences between the two sexes in the health status [22]. The data of the present study are part of a larger study of the blood proteomic changes in T2DM, where several proteins were identified to be differentially expressed in T2DM, mostly were upregulated [6]; however, the influence of gender on T2DM and other disorders cannot be corrected for by the traditional proteomic statistical analysis. Therefore, in this study, we compared the serum protein profile of healthy men and women who were included in the main study.

## 2. Materials and Methods

### 2.1. Study Subjects

A subset of samples was obtained from 6 healthy subjects, 3 males/3 females, aged 41, 42, and 47 years (age and sex matched), selected from a larger number of Bahraini volunteers involved in the study of T2DM biomarkers, between September 2014 and February 2015 (Table 1). The main inclusion criteria were original Bahraini national, nondiabetic, apparently healthy, male or female, and willing to participate in the study. Exclusion criteria included younger (<40 years) and relatively older (>50 years) ages, acute or chronic disorders, prolonged treatment, e.g., antibiotics and immune suppressive drugs. Informed consent was obtained from each study subject before blood collection. The study design followed the Helsinki Declaration terms. The study was approved by the Research and Ethics Committees of the Arabian Gulf University (AGU) and Salmaniya Medical Complex Hospital (SMC), Manama, Bahrain.

#### 2.1.1. Sample Collection

Approximately 10 ml of venous blood sample was collected from each study subject, 10–12 hours after overnight fasting, into different collection tubes (Thermo Fisher Scientific, Massachusetts, U.S.A.), and centrifuged (3000 × g for 10 min) to collect the plasma and serum.

### 2.2. Biochemical Tests

#### 2.2.1. Glycemic Profile

The fasting blood glucose (FBG) level was analyzed by Clinical Analyzer ROCHE COBAS INTEGRA 800 (Rotkreuz, Switzerland), and the results were expressed as mmol/L. The glycated hemoglobin (HbA1c) was measured by Clinical Chemistry analyzer (Beckman Coulter, AU, USA), and the HbA1c to total Hb ratio (HbA1c %) was calculated.

#### 2.2.2. Serum Lipid Profile

The lipid profile parameters: total cholesterol, low-density lipoprotein cholesterol (LDL-C), high-density lipoprotein cholesterol (HDL-C), and triglycerides (TGs), were measured in the biochemistry laboratory at SMC, using Clinical Analyzer ROCHE COBAS INTEGRA 800 (Rotkreuz, Switzerland).

### 2.3. Protein Profiling Using LC-MS/MS

The protein profile analysis was done by LC-MS/MS as previously described [23, 24], in brief;

#### 2.3.1. Serum Preparation - Protein Depletion

Pierce albumin/IgG removal kit (Thermo Scientific, U.S.A.) was used to remove the major subclasses of gamma globulin (IgG) and human serum albumin (HSA) from serum as the most abundant serum proteins, in order to detect the least abundant ones (depleted serum). The unprocessed serum (nondepleted serum) was also used. Thereafter, the exact quantity of proteins in serum was determined in order to calculate the amount of sample needed for analysis.

#### 2.3.2. In-Solution Protein Digestion

The serum samples, both nondepleted and depleted, were diluted 1: 1 with 0.1% *Rapi*Gest™ SF (Waters, UK). Thereafter, the sera were pooled into 2 sets of samples, 3 healthy males and 3 healthy females. The total load for each pooled sample for analysis was 100 *μ*g of protein/peptide (PPs) in a final volume of 25 *μ*l. Then, the PPs in the pooled samples were denatured in a Thermo mixer R (Eppendorf, Hamburg, Germany) at a speed of 8 × g at 80°C for 15 min. In the end, the PPs were digested by trypsin at a 1 : 50 ratio (Promega Corporation, Madison, WI, USA). The PPs digests were then analyzed using Synapt G2 MS (Waters, Manchester, UK).

#### 2.3.3. Protein Identification

The 1-dimensional Nano Acquity liquid chromatography coupled with tandem mass spectrometry on a Synapt G2 instrument (Waters, Manchester, UK) was used for label-free quantitative expression protein profiling. For ESI mass spectrometry analyses, the instrument settings were optimized on the MassLynx tune page. A protein digest of 3-*μ*g was loaded on the column, and the samples were spiked with yeast alcohol dehydrogenase (ADH, P00330) as an internal standard to digest to give 200 fmol per injection for absolute quantitation. An Acquity sample manager was used for the injection of the analyzed samples.

The samples were analyzed in duplicate runs, and data were acquired using the MassLynx program (version. 4.1, SCN870; Waters) operated in resolution and positive polarity modes. The acquired MS data were background subtracted, smoothed, and deisotoped at the medium threshold. Progenesis QI V2.0 (QIfp) for proteomics (Nonlinear Dynamics/Waters, UK) was used for automated data processing and database searching. The generated peptide masses were searched against in UniProt species-specific protein sequence database using the Progenesis QI V2.0 (QIfp) for proteomics for protein identification and quantification (Nonlinear Dynamics/Waters, UK).

### 2.4. Data Analysis and Informatics

#### 2.4.1. Proteomics Data

Progenesis QI V2.0/TransOmics Informatics (Waters Scientific, Manchester, UK) software was used to process and search the data using the principle of search algorithm as previously described [24, 25]. The data were filtered to show only statistically significant differences (*p* < 0.05, ANOVA) coupled with a change in proteins' abundance by 1.5 fold or more (Maximum Fold Change—MFC ≥1.5). Additionally, the absolute quantification was performed using ADH as an internal standard to give an absolute amount of each identified protein/peptide.

#### 2.4.2. Bioinformatics

For gene localization, PPs identification and classification, sites of expression in human tissues, biological and molecular functions, and related genetic disorders, several proteomic, genomic, and informatics databases were searched. These included https://www.uniprot.org; https://www.ncbi.nlm.nih.gov/gene; https://www.genecards.org/; https://www.omim.org/; https://www.unicarbkb.org/ (for glycated proteins only). Gene expression databases were approached through https://www.uniprot.org/uniprot/. Other information was obtained from published data through https://www.ncbi.nlm.nih.gov/pubmed and Google as nonprofessional site.

## 3. Results

### 3.1. Description of the Differentially Expressed Proteins in Healthy Men and Women

As seen in Table 2, a total of 20 PPs were found to be differentially expressed between men and women. Using the proteomic databases, most of the identified PPs are expressed in several tissues and organs; however, the following organs/tissues are the predominant producers of the identified PPs; the liver (9 PPs), immune cells, mostly lymphocytes (5 PPs), gastrointestinal tract (GIT) including the appendix, oral cavity and gallbladder (4 PPs), female genital organs (3 PPs), and the central nervous system, specifically, the corpus callosum (1 PP). Worth noting, some PPs were expressed predominantly in more than one tissue. Based on function, the PPs were broadly classified into the following categories: (a) lipid metabolism (5 PPs), (b) immunity (4 PPs), (c) connective tissue (3 PPs), (d) gene expression (3 PPs), (e) acute phase proteins (2 PPs), (f) transport proteins (1 PP), (g) signal transduction (1 PP), (h) unknown (1 PP). However, many PPs could be assigned to more than one class (source, e.g., using Reactome pathway database https://reactome.org or Panther classification system https://www.pantherdb.org/).

### 3.2. Abundance of Upregulated PPs in Healthy Women and Men

**Figure 1:**
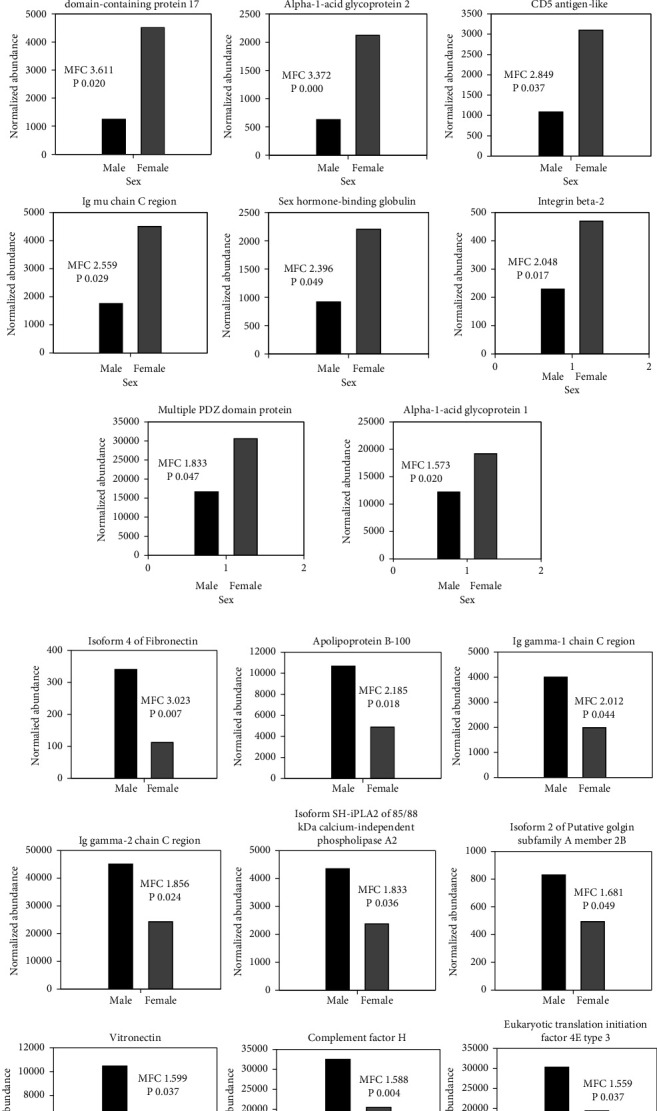
Histograms of the normalized abundance of differentially expressed proteins/peptides (PPs) identified in healthy males (black) and females (grey). (a) PPs upregulated in women compared with men, and (b) PPs upregulated in men compared with women. The *P*-values (ANOVA) and magnitude of change (maximum fold change–MFC) are indicated for each PP in each figure. The proteins were identified using label-free quantified liquid chromatography-tandem mass spectrometry on Synapt G2 analysis–LC-MS/MS. Progenesis QI for proteomics software was used for data analysis. *Note*. There are no bar errors as samples are usually pooled in LC-MS/MS analysis.

Figure 1 shows the differentially expressed PPs, the PPs which were upregulated in women (A), and the ones which were upregulated in men (B). The differences in the abundance of PPs in the serum between the two sexes were rated by the maximum fold change (MFC). The PPs, which were increased by more than 3 folds, i.e., MFC >3, were mostly upregulated in women. These PPs in decreasing order were apolipoprotein C-II (8.6 folds), homeobox protein Hox-D13 (6.0 folds), apolipoprotein A-II (4.0 folds), isoform 4 of coiled-coil domain-containing protein 17 (3.6 folds), and alpha-1-acid glycoprotein 2 (3.4 folds). However, in men, only the isoform 4 of fibronectin was increased by more than 3 folds compared with women (MFC 3.02). For the fold changes and *p*-values, see Table 2 and Figure 1.

### 3.3. Identification of Differentially Expressed Genes: Bioinformatics Data Extraction

Bioinformatic analyses were applied to determine the differentially expressed genes of the identified PPs, using the accession numbers obtained from LC-MS/MS analysis. The genetic background of the identified PPs was obtained by navigation through Protein knowledgebase–UniProtKB site: https://www.uniprot.org/uniprot/. All identified PPs were exclusively expressed by genes (gn) located in autosomal chromosomes (Ch), as follows: Ch 1 (4 gn), Ch 2 (3 gn), Ch 9 (3 gn), Ch 14 (3 gn), Ch 17 (2 gn), and Chs 3, 12, 19, 21, and 22, each (1 gn). The genes, Ch, and the gene locus in the Ch are shown in Table 2.

### 3.4. Proteins/Peptides Upregulated in Healthy Women

**Figure 2:**
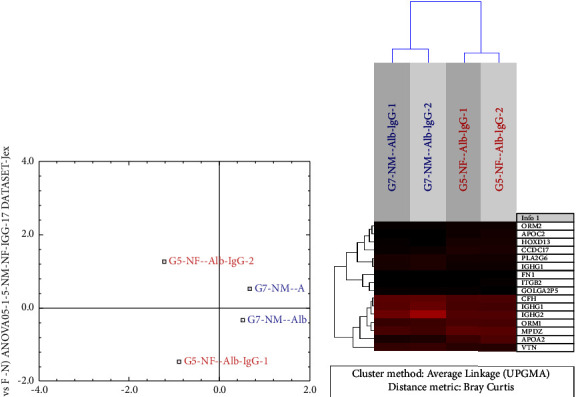
(a) Correspondence analysis (CA) plot of healthy subjects' nondepleted serum using the expression dataset of 15 identified proteins/peptides (PPs) that were significantly differentially expressed (*P* < 0.05-ANOVA, MFC (minimum fold change) ≥1.5-) between men and women. The expression profiles of the identified PPs distinctively differentiate women from men using correspondence analysis. The letters in the background are the accession numbers of all the identified PPs in the analysis. (The image was generated using J-express pro V1.1 software program (java.sun.com)). (b) Unsupervised hierarchical cluster analysis of the expression profiles of nondepleted serum samples using 17 PPs that differ significantly (*P* < 0.05-ANOVA, MFC ≥1.5) between men (blue) and women (red). The dendrogram was generated using the Bray–Curtis correlation distance metric and an average linkage clustering method from the J-express pro V1.1 software program (java.sun.com). *Note*. One PP (IGHG1) had 2 accession numbers (subtypes of the same PP) are shown in the figure as two different PPs.

**Figure 3:**
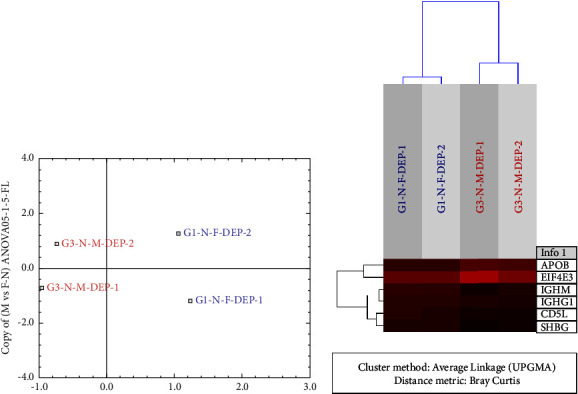
(a) Correspondence analysis (CA) plot of depleted serum samples from healthy subjects using the expression dataset of 5 identified proteins/peptides (PPs) that were significantly differentially expressed (*P* < 0.05-ANOVA, MFC ≥1.5- minimum fold change) between men and women. The expression profiles of the identified PPs clearly differentiate women from men using correspondence analysis. (The image was generated using J-express pro V1.1 software program (java.sun.com)). (b) Unsupervised hierarchical cluster analysis of the expression profiles of depleted serum samples using 5 proteins that differ significantly (*P* < 0.05, analysis of variance; maximum fold change ≥1.5) between men (red) and women (blue). The dendrogram was generated using the Bray–Curtis correlation distance metric and an average linkage clustering method from the J-express pro V1.1 software program (java.sun.com). *Note*. One PP (IGHG1) was upregulated in both nondepleted (Figure 2) and depleted sera.

As shown in Figure 1(a), 11 PPs were upregulated in healthy women compared with men (ANOVA <0.05, MFC >1.5); 8 were identified in nondepleted sera (Figures 2(a) and 2(b)) and 3 in depleted sera (Figures 3(a) and 3(b)). The expression profiles of the identified PPs in nondepleted serum (Figure 2(a)) and depleted serum (Figure 3(a)) were strongly differentiated between women and men using correspondence analysis. The hierarchical cluster analysis of the expression profiles of the identified PPs in nondepleted sera is shown in (Figure 2(b)), and that of the PPs in depleted sera is shown in Figure 3(b), which both differentiated between men and women. Of these PPs, the ones which were expressed predominantly in the liver are 4 PPs, in lymphocytes and bone marrow cells were 3 PPs and 1 PP in the CNS (corpus callosum), while only 2 PPs were expressed predominantly by females' genital organs. The classes of PPs upregulated in both women and men were the lipid metabolism and immunity classes, and in women, the 2 classes included the following PPs: apolipoprotein A-II, apolipoprotein C-II and CD5 antigen-like, and Ig mu chain C. The acute phase proteins (alpha-1-acid glycoprotein 1 and alpha-1-acid glycoprotein 2) and signal transduction (multiple PDZ domain protein) classes of PPs were upregulated exclusively in women. Furthermore, 2 PPs are involved in gene expression; the homeobox protein Hox-D13 and isoform 4 of coiled-coil domain-containing protein 17, and one PP (integrin beta-2) classified as connective tissue and another as transporter (sex hormone-binding globulin) PP, were upregulated also in women.

### 3.5. Proteins/Peptides (PPs) Upregulated in Healthy Men

The PPs upregulated in men compared to women were 9 PPs (Figure 1(b)), 7 were identified in nondepleted serum while 2 were identified in depleted serum. Using the correspondence analysis, the expression profiles of the former (Figure 2(a)) and the latter (Figure 3(a)) PPs strongly differentiated men from women. As shown in Figures (2(b) and 3(b)), the hierarchical cluster analysis of the expression profiles of the 7 PPs in the nondepleted and that of the 2 PPs in depleted sera, respectively, clearly distinguished between men and women. Three of the upregulated PPs in men, the complement factor H, Ig gamma-1 chain C region, and Ig gamma-2 chain C region, belong to the immunity class of PPs. Two PPs were classified under each of the lipid metabolism (Apo-B100 and isoform SH-iPLA2 of 85/88 kDa calcium-independent phospholipase A2) and connective tissue (isoform 4 of fibronectin and vitronectin) classes. One PP, the eukaryotic translation initiation factor 4E type 3 (fragment), is involved in gene expression, specifically translation. However, one PP, the isoform 2 of putative golgin subfamily A member 2B, is likely to be allocated to Golgi apparatus, but its class is unknown.

### 3.6. Ingenuity Pathway Analysis (IPA)

**Figure 4:**
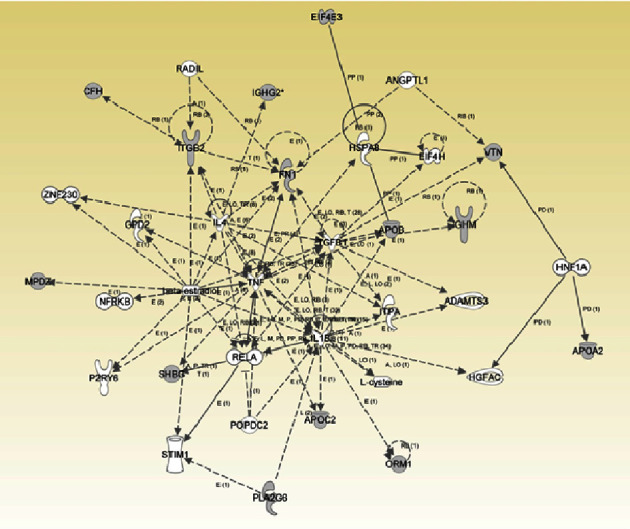
Pathway analysis of network signaling of 32 proteins represented in the ingenuity pathway analysis database included 14 PPs identified in this study (grey shading). Only thirty-two (32) of the 100 proteins were mapped in the IPA database and were implicated in the pathway analysis of multiple signaling networks including cell-to-cell signaling and interaction, cell death and survival, cellular movement, and immune cell trafficking (the image was generated using ingenuity pathway analysis program (IPA version 49:309) https://qiagen.force.com).

The ingenuity pathway analysis (IPA) is used for meaningful interpretation of gene expression data using prior biological knowledge. For networking of the identified PPs between each other and other PPs sharing the same function, and for casual and functional relation to disease and immunity, the 20 PPs were subjected to IPA, using an all-in-one, web-based software application, from QIAGEN Bioinformatics. Only 14 PPs (7 were upregulated in men and 7 in women) (Table 3) were mapped in the IPA database together with another 18 PPs not identified in this study (Figure 4). These PPs were found to be implicated in several networks. The gene sources of these proteins were highlighted in grey in Figure 4. The cellular localization of some of these PPs included the plasma membrane, cytoplasm, nucleus, and extracellular space (Table 3).

## 4. Discussion

The fact that men and women biologically, morphologically, and physiologically are different [26] is inspiring to study the nature and magnitude of this difference at the molecular level. It is well known that genomically and genetically both sexes possess the same chromosomes and genes except for the sex chromosome (XY), namely the Y chromosome genes, e.g., *SRY* gene [27]. In this study, proteomic analysis revealed marked differences in the levels of 20 PPs, none of which is encoded by genes located in the sex chromosomes, controversial to what was observed elsewhere [28]. Furthermore, the PPs upregulation was more evident in women, confirming a recent report showing significant sexual dimorphism in protein abundance between twin pairs of the opposite sex [28], although men have the unique chromosome Y.

The observation that all identified PPs are expressed by genes located in the autosomal chromosomes is supported by a previous study reporting that the sex differences in part are due to differences in the expression of genes not in sex chromosomes [29]. Furthermore, 3 PPs known to be regulators of gene expression were differentially expressed in this study, supporting the sex-biased expression independent of the sex chromosomes. Two transcription factors, homeobox protein Hox-D13 and isoform 4 of coiled-coil domain-containing protein 17 (CCDC17), were upregulated in women, while a translational factor, eukaryotic translation initiation factor 4E (eIF4E) type 3 (Fragment), was upregulated in men. The Hox gene family products are known to be transcriptional factors involved in female reproductive system development and function [30]. However, the other 2 PPs are not known to have any sex-specific role. The only PPs in this study that was directly related to sex were the sex hormone-binding globulin (SHBG), which was upregulated in women. One study showed that SHBG mRNA is strongly correlated with serum SHBG protein level, with higher levels of mRNA and protein in women than in men [31], in line with the present study.

Of the markedly differentially expressed PPs, 11 were upregulated in women and 9 in men; however, the differences in abundance were more marked in the PPs upregulated in women. In this study, the experimental system was set to select only markedly differentially expressed PPs; however, in biological systems, function and homoeostasis depend on a fine balance of molecules, i.e., not necessarily the quantity [32, 33]. Noticeably, was the upregulation of Apo AII and Apo CII in women, and that of Apo B100 in men. The former two were the predominant Apo proteins in chylomicron and VLDL, which are not involved directly in atherosclerosis and coronary heart disease (CHD), while Apo B100, the major protein in LDL, is the carrier of pathological cholesterol [34, 35]. It is well known that men are more susceptible to CHD compared to women of the same age before menopause [8], which is consistent with the present finding.

The sex-based immunological dimorphism was known a long time ago [36]; however, detailed analysis showing the abundance of immunoglobulins (Ig) chains at the transcriptome or proteome level was rarely investigated. In this study, the upregulation of Ig mu chain C region (IgM heavy chain) in women and that of Ig gamma-1 and 2 chain regions (IgG heavy chains) and complement factor H (the complement alternative pathway) in men probably reflects the difference between the two sexes in response to antigenic challenges. In line with our findings are Saudi and Iranian studies, both showed higher levels of IgM in healthy women compared with men and higher IgG in men compared with women in the Saudi but not in the Iranian study [37, 38]. Studies of sex differences in immune responses following infection/vaccination showed that women across all age groups were able to mount higher immune responses to infections and vaccines than men [14, 39]. In contrast, women compared to men are at much higher risk for development of autoimmune diseases, e.g., SLE [40].

To the best of our knowledge, the differences in the blood levels of the acute phase proteins, alpha-1-acid glycoprotein-1 and 2 (AGP1 and 2), also known as orosomucoid, between men and women were not reported before. In this study, both PPs were upregulated in women. The associations of AGPs with rheumatoid arthritis [41] and other autoimmune diseases, e.g., Grave's disease [42], together with the high prevalence of these disorders in women [11], are strongly supporting our findings. For signal transduction PPs, the multiple PDZ domain protein, which is known to have the highest expression level in the corpus callosum (brain), was the only PP identified in this study. It is involved in the control of cell polarity and signal transduction [43, 44], and it is also known to be associated with several and diverse pathologies [45], thus, its upregulation in women is worth further investigation.

In this study, the upregulation of the PPs in men was not as high as in women in terms of abundance (fold change). The upregulation of the 2 connective tissue (CT) proteins, fibronectin and vitronectin in men, is not unexpected since men and women have different musculoskeletal system properties [46]. The sex difference in the levels of both PPs was not reported before; however, both proteins were shown to be upregulated in T2DM [6, 47]. The differences in CT proteins between the 2 sexes have clinical implications; e.g., the incidence of anterior cruciate ligament injury is almost 10 times higher in women compared with men performing the same activity [48], an observation that supports our findings.

The last PP that was upregulated in men is the isoform 2 of putative golgin subfamily A, member 2B, one of the Golgi apparatus proteins, which is probably encoded by a pseudo-gene (https://www.genecards.org/cgi-bin/carddisp.pl?gene=GOLGA2P5). However, no single article was published about this PP in the PubMed database. This and other PPs listed in Table 2 are not discussed here but are worth further investigation.

As a summary of the above, we further explored the functional characteristics and relatedness of the 20 PPs with disease and other immune-mediated disorders using ingenuity pathway analysis (IPA). The principle of the IPA was explained previously [49]. Only 14 of the 20 PPs were mapped in the IPA database and were found to be implicated in multiple signaling networks including cell-to-cell signaling and interaction, lipid metabolism, and small molecule biochemistry. The functional annotation of these proteins with others as transporters, transmembrane receptors, and catalysts is referred to in Table 3; moreover, these networks are in connections with different drug agents (data not shown).

Of the limitations of this study is the small sample size, and lack of validations of the LC-MS/MS results with other methods, although results were previously validated by testing 3 PPs including fibronectin in 80 plasma samples by ELISA [47].

In conclusion, 20 PPs were found to be differentially expressed in healthy men and women, 11 upregulated in women with high fold change, while 9 were upregulated in men with lower fold change compared to women. Although differentiating between the two sexes, none of the identified PPs is encoded by genes allocated to sex chromosomes. The frequently obvious links between sex and PPs upregulation, and the physiological and pathological roles of the latter are validation for this proteomic data; however, further investigation is needed. The differences between men and women at the molecular level are crucial for a better understanding of sex and gender-related disorders which is the first step towards precision/personalized medicine. Although the biological differences between men and women were thoroughly studied, to the best of our knowledge, this is the first study in humans' proteomics for the same purpose.

## Figures and Tables

**Table 1:** Clinical, biochemical, and demographic characteristics of the study subjects.

Serial number	Sample ID	Clinical diagnosis	Sex	Age (years)	Glucose (mmol/l)	HbA1c (%)	LDLC (mmol/l)	HDLC (mmol/l)	TGs (mmol/l)	T CHOL (mmol/l)
1	S4	Healthy	F	42	5.7	ND	3.3	1.5	1.2	5.3
2	S52	Healthy	M	42	5.1	ND	2.5	0.98	1.4	4.1
3	S8	Healthy	F	41	5.4	ND	2.5	1.7	1	4.7
4	S15	Healthy	M	41	4.9	ND	3.1	1.2	0.4	4.5
5	SR	Healthy	F	47	4.9	ND	3.73	1.6	0.8	5.7
6	SK	Healthy	M	47	5.5	ND	3.2	0.9	1.7	4.3

LDLC, low-density lipoprotein cholesterol; HDLC, high-density lipoprotein cholesterol; TGs, triglycerides; T CHOL, total cholesterol; ND, not done; HbA1c, blood glucose and glycated hemoglobin; T2DM, type 2 diabetes mellitus; M, male; F, female.

**Table 2:** List of upregulated proteins/peptides (PP)s in healthy men (grey shadow) and women, showing the PP category, accession number, gene, and gene chromosomal location.

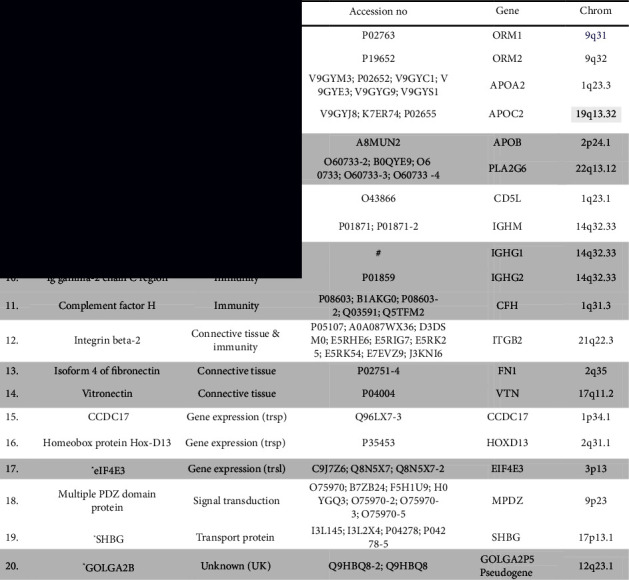

^*∗*^PLA2G6: isoform SH-iPLA2 of 85/88 kDa calcium-independent phospholipase A2. ^*∗*^CCDC17: isoform 4 of coiled-coil domain-containing protein 17. ^*∗*^eIF4E3: eukaryotic translation initiation factor 4E type 3 (fragment). ^*∗*^SHBG: sex hormone-binding globulin. ^*∗*^GOLGA2B: isoform 2 of putative golgin subfamily A member 2B. trsp: transcription. trsl: translation. #: A0A087WV47; A0A087WYC5; A0A087WYE1; A0A087X010; A0A0A0MS07; A0A0A0MS08; P01781; P01857.

**Table 3:** List of the 14 identified protein/peptides that were implicated in the ingenuity pathway analysis database.

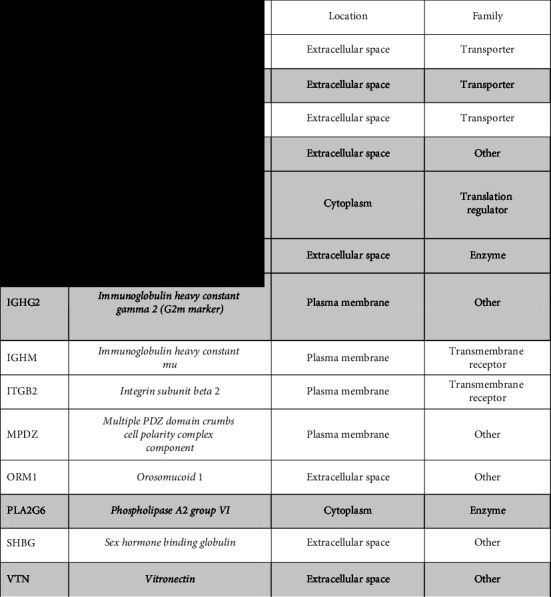

Grey filling indicates PPs upregulated in men.

## Data Availability

The datasets used and/or analyzed during the current study are available from the corresponding author upon reasonable request.
